# Supplementary figures and images for: Cortico-autonomic local arousals and heightened somatosensory arousability during NREMS of mice in neuropathic pain
Source: eLife. 2021 Jul 6;10:e65835. doi: 10.7554/eLife.65835 (PMC8291975; doi:10.7554/eLife.65835)

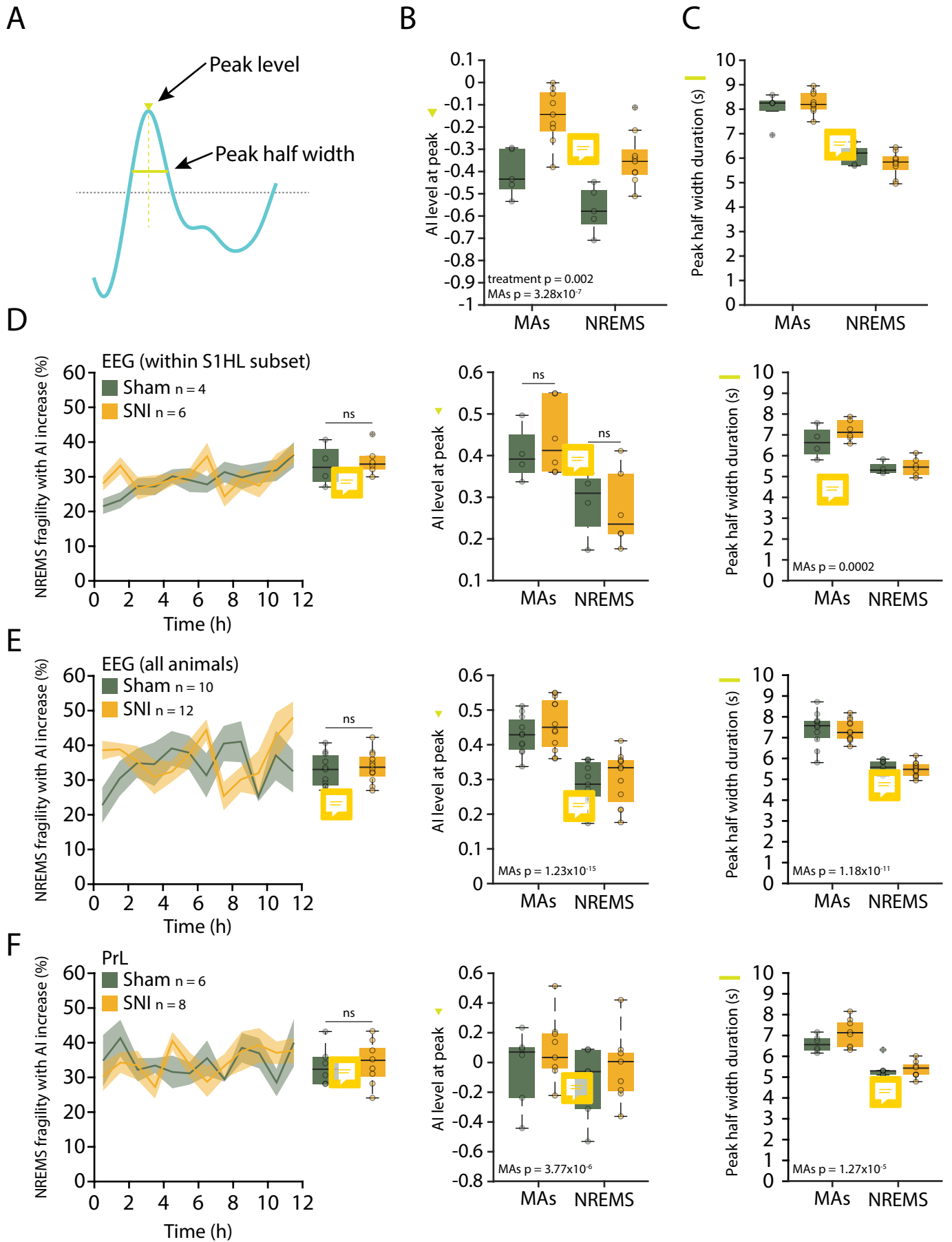

Supplement: Figure 1—source data 1. — There is one .csv file for each panel except (G). In case of the two datasets used in panel (G), one .csv file for each dataset is generated and appropriately labeled. Additionally, a commented .pdf file with test statistics and p values is provided. [file elife-65835-fig1-data1.zip › Figure_6_figure_supplement_1/Figure_6_figure_supplement_1.pdf]

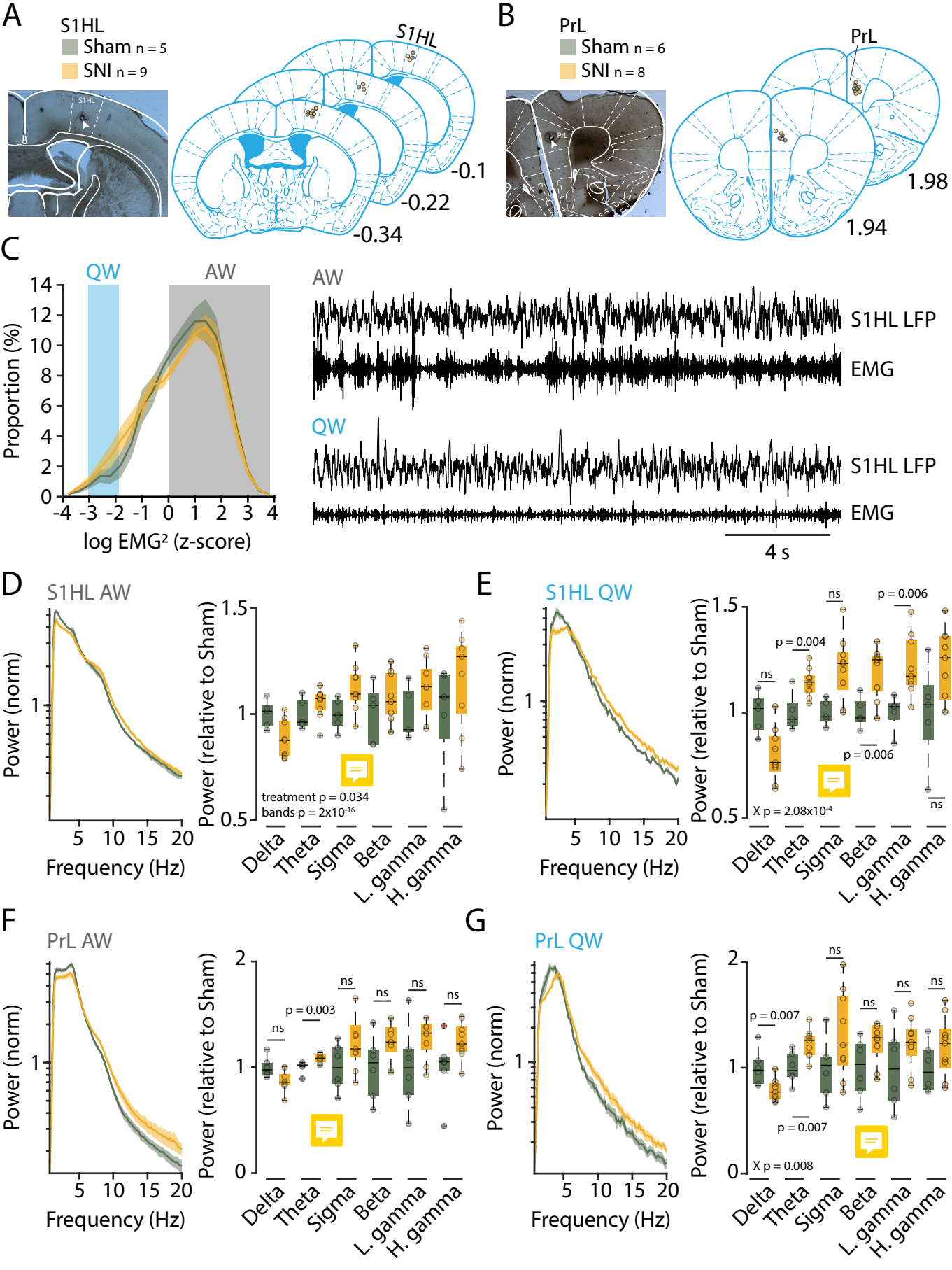

Supplement: Figure 2—source data 1. — There is one .csv file for each panel. Additionally, a commented .pdf file with normality test, test statistics, and p values is provided. [file elife-65835-fig2-data1.zip › Figure_2/Figure_2.pdf]

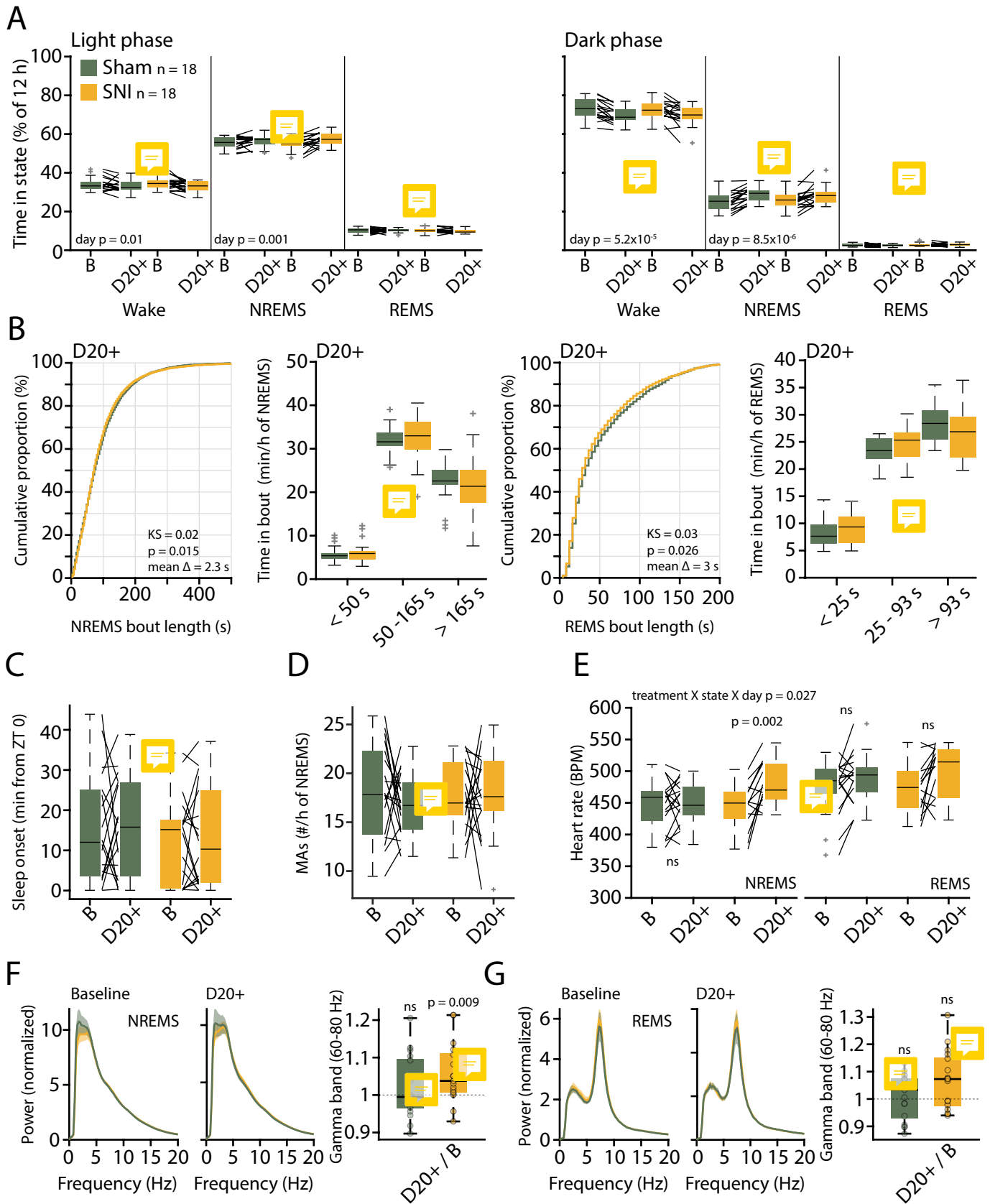

Supplement: Figure 3—source data 1. — For panels (A, C, D), two .csv files containing data from all three panels are provided for the light and the dark phase. For panel (B), six .csv files are provided for each dataset in the cumulative plots and the binned data for both non-rapid eye movement sleep (NREMS) and rapid eye movement sleep (REMS). One .csv file is provided for panel (E), whereas for panels (F, G), two .csv files are included separately for the power spectra at baseline and at D20+. One .csv file is provided for the gamma power analysis combining both NREMS and REMS. Additionally, a commented .pdf file with test statistics and p values is provided. [file elife-65835-fig3-data1.zip › Figure_3/Figure_3_SleepInSNI.pdf]

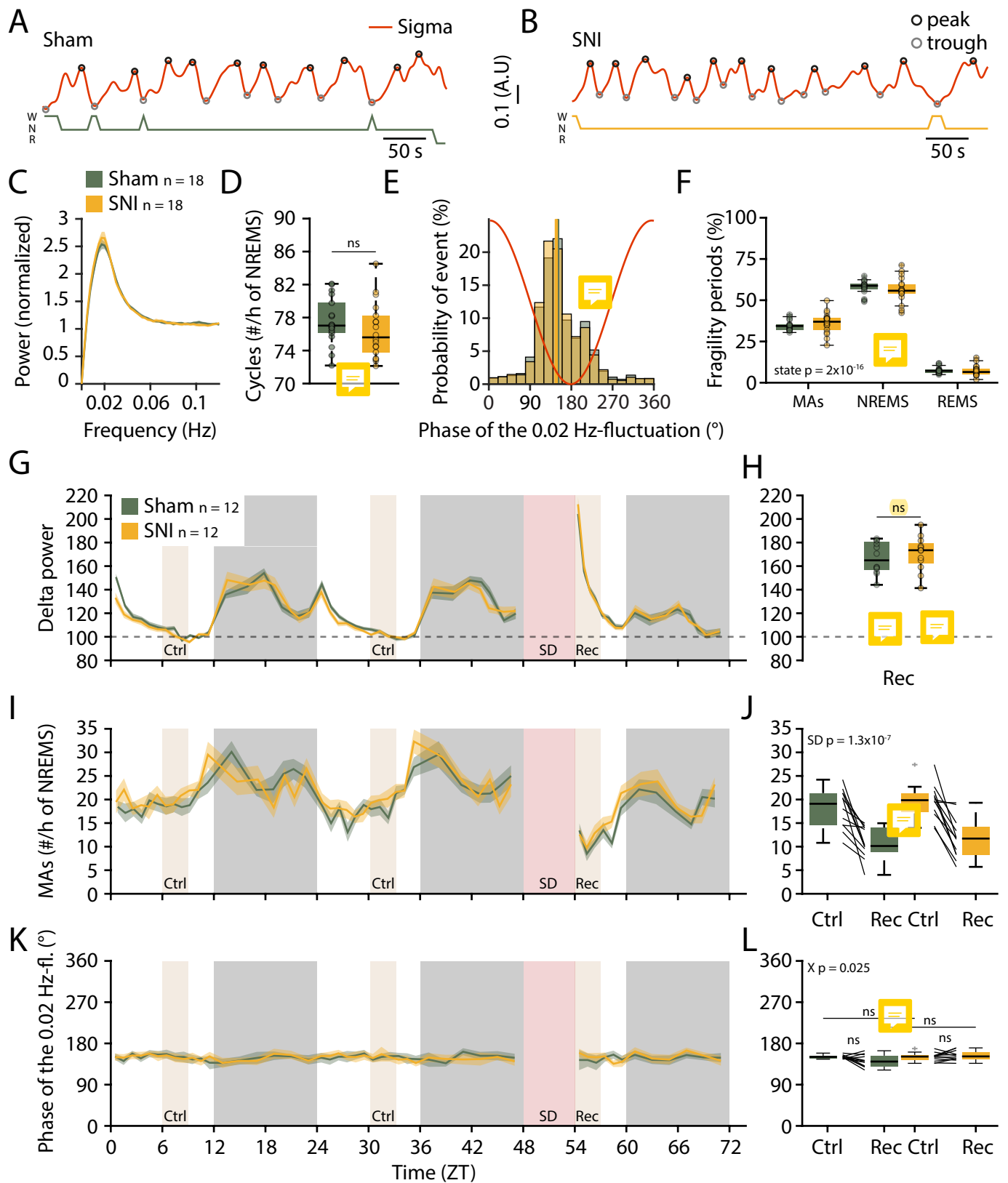

Supplement: Figure 4—source data 1. — There is one .csv file for each panel. Additionally, a commented .pdf file with normality test, test statistics, and p values is provided. [file elife-65835-fig4-data1.zip › Figure_4/Figure_4.pdf]

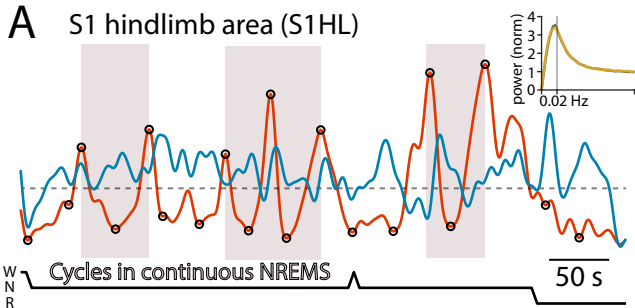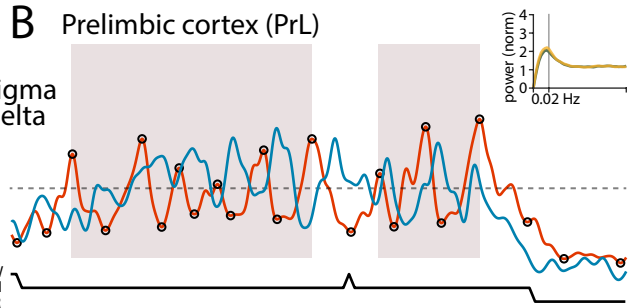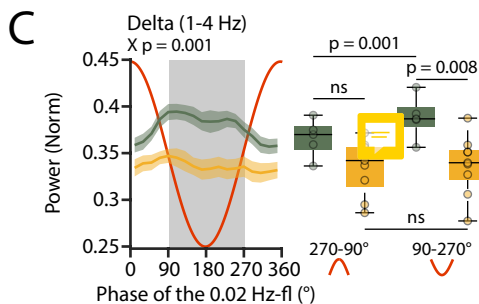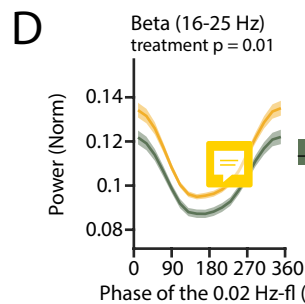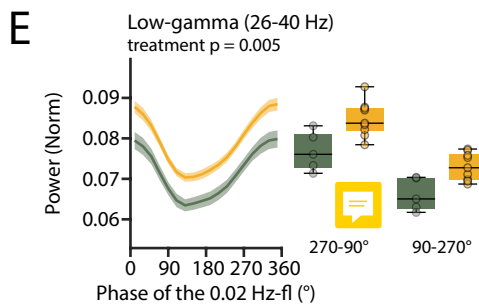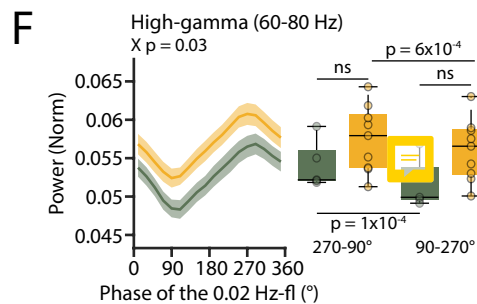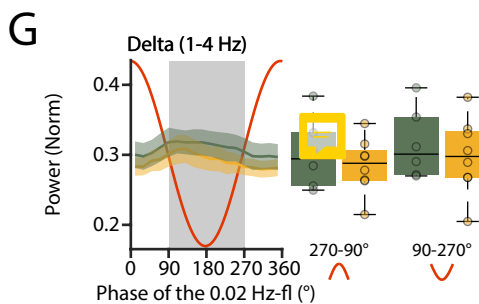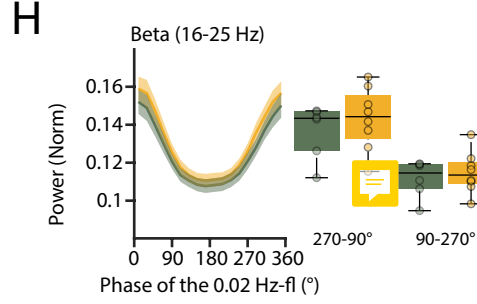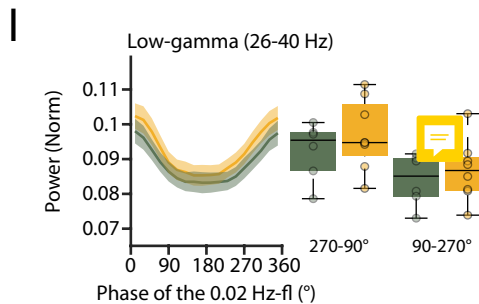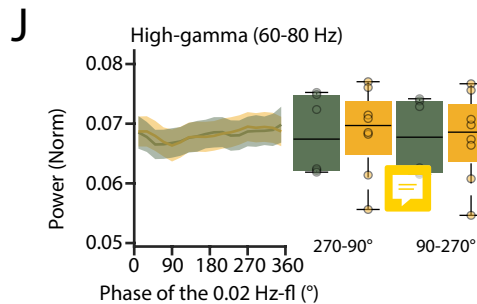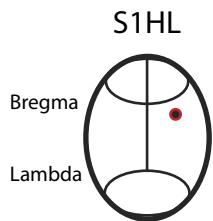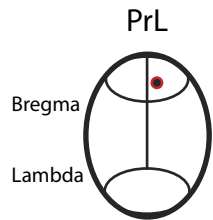

Supplement: Figure 5—source data 1. — There is one .csv file for each panel. Additionally, a commented .pdf file with normality test, test statistics, and p values is provided. [file elife-65835-fig5-data1.zip › Figure_5/Figure_5.pdf]

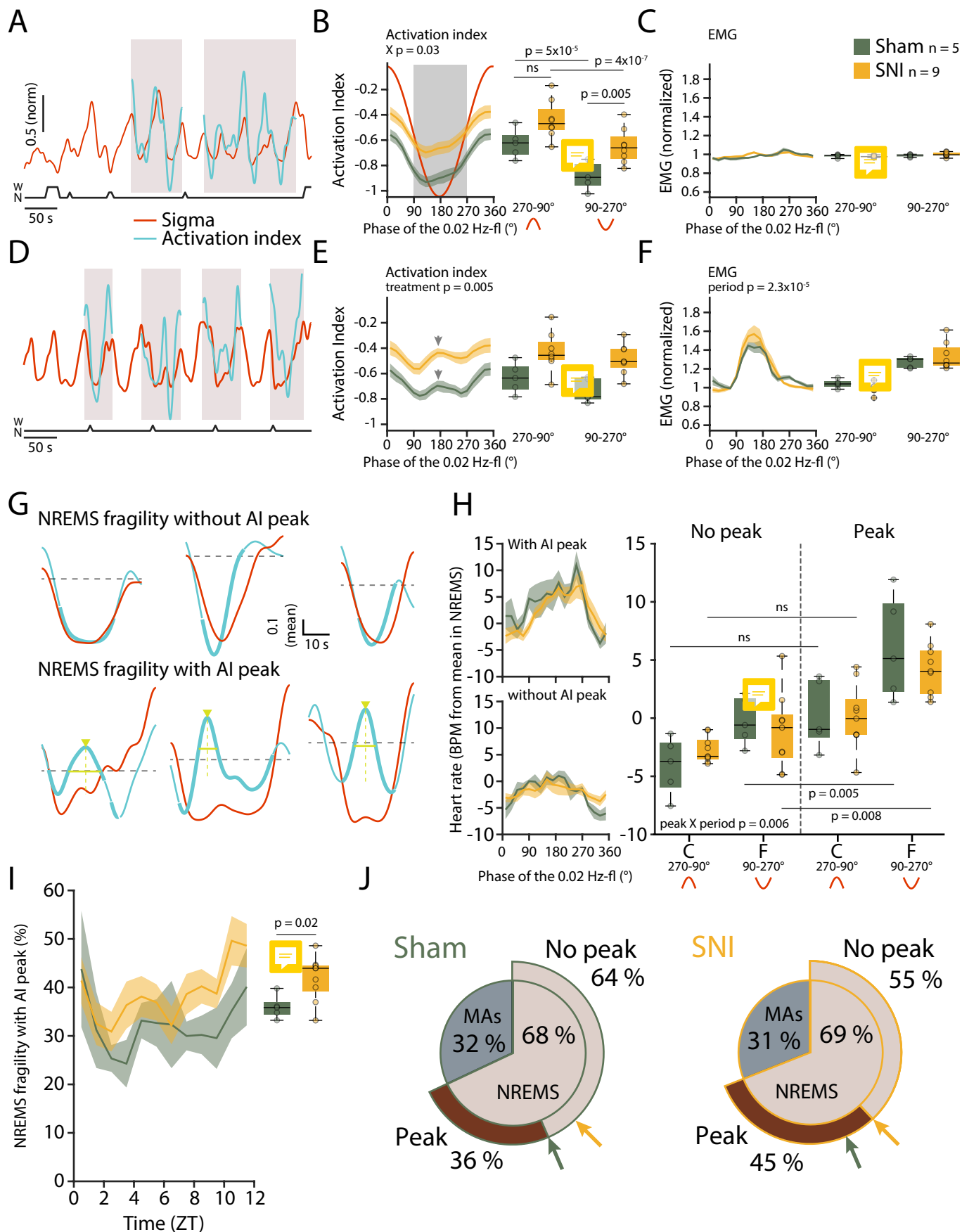

Supplement: Figure 6—source data 1. — There is one .csv file for each panel. Additionally, a commented .pdf file with normality test, test statistics, and p values is provided. [file elife-65835-fig6-data1.zip › Figure_6/Figure_6.pdf]

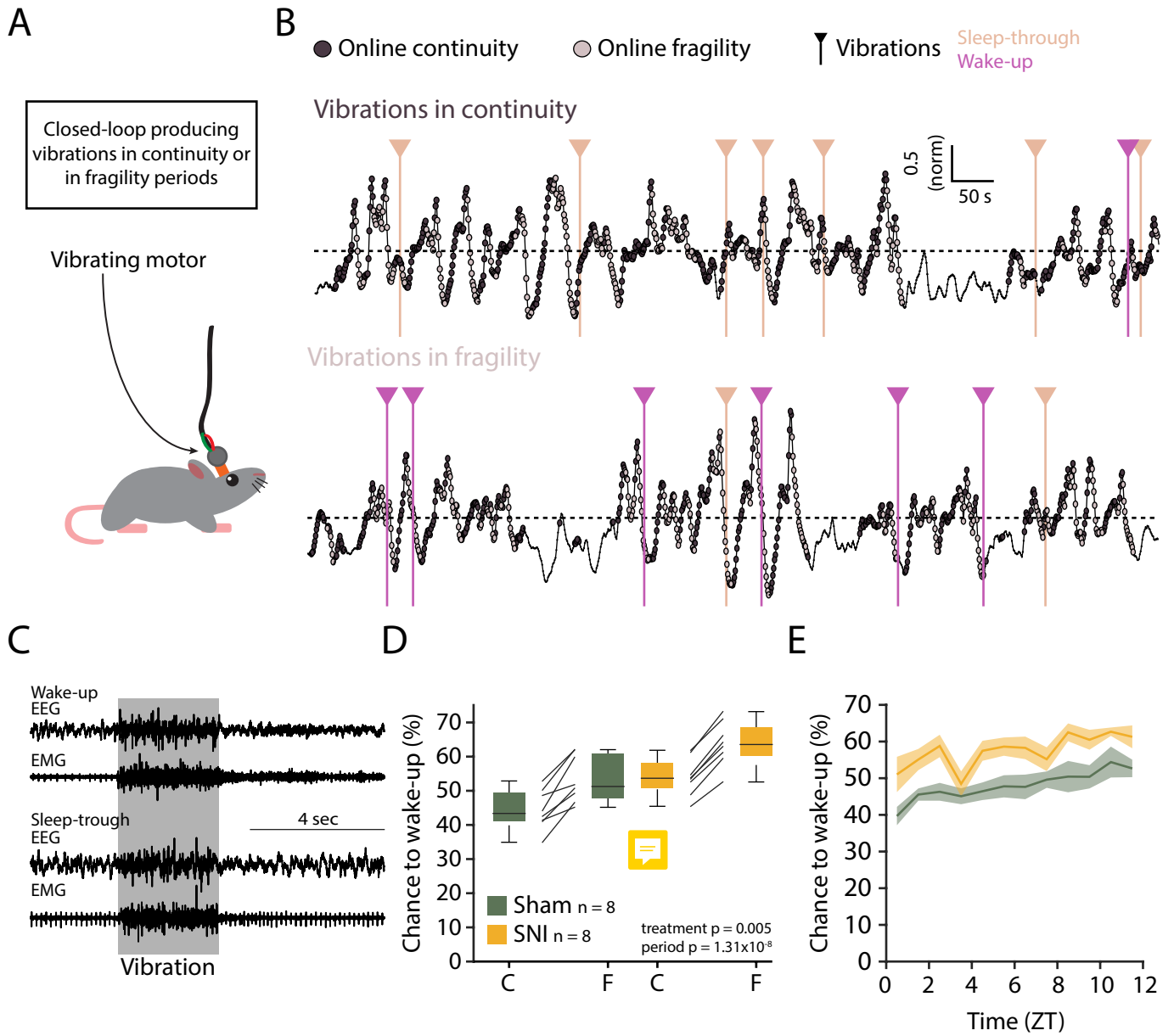

Supplement: Figure 8—source data 1. — There is one .csv file for each panel. Additionally, a commented .pdf file with normality test, test statistics, and p values is provided. [file elife-65835-fig8-data1.zip › Figure_8/Figure_8.pdf]
