# Supplementary material for: Cortico-autonomic local arousals and heightened somatosensory arousability during NREMS of mice in neuropathic pain
Source: eLife. 2021 Jul 6;10:e65835. doi: 10.7554/eLife.65835 (PMC8291975; doi:10.7554/eLife.65835)
Supplement: Supplementary file 1. — We have instead removed this info from all figure legends to simplify reading. [file elife-65835-supp1.docx]

Supplementary Table for Statistics for every figure panel.

| **Figure** | **Test type** | **N** | **Stat-value** | **P-value** | **Cohen’s effect-size** |
| --- | --- | --- | --- | --- | --- |
| 1D-1 | Rayleigh | 9476 | z = 4214.7 | p < 1x10^-16^ | / |
| 1D-2 | Rayleigh | 9476 | z = 3489 | p < 1x10^-16^ | / |
| 1D-3 | Rayleigh | 9476 | z = 1416 | p < 1x10^-16^ | / |
| 1D-4 | Rayleigh | 9476 | z = 548.16 | p < 1x10^-16^ | / |
|  |  |  |  |  |  |
| 2D | Mixed-model ANOVA | Sham = 5  SNI = 9 | Treatment:  F_(1,12)_ = 5.745  Frequency bands:  F_(1,12)_ = 1578.2  Interaction:  F_(5,60)_ = 1.75 | Treatment:  p = 0.034  Frequency bands:  p =2x10^-16^  Interaction:  p = 0.13 | Treatment:  f = 0.69  Frequency bands:  f = 11.47  Interaction:  Cohen f = 0.38 |
| 2E | Mixed-model ANOVA | Sham = 5  SNI = 9 | Treatment:  F_(1,12)_ = 21.75  Frequency bands:  F_(1,12)_ = 2386.18  Interaction:  F_(5,60)_ = 5.76 | Treatment:  p = 5.4 x10^-4^  Frequency bands:  p =2x10^-16^  Interaction:  p = 2.08x10^-4^ | Treatment:  f = 1.35  Frequency bands:  f = 14.1  Interaction:  f = 0.69 |
| 2E - Delta | Unpaired t-test | Sham = 5  SNI = 9 | t_(12)_ = 2.96 | p = 0.012 | d = 1.76 |
| 2E - Theta | Unpaired t-test | Sham = 5  SNI = 9 | t_(12)_ = -3.54 | p = 0.004 | d = -1.85 |
| 2E - Sigma | Unpaired t-test | Sham = 5  SNI = 9 | t_(12)_ = -3.1 | p = 0.009 | d = -1.9 |
| 2E - Beta | Unpaired t-test | Sham = 5  SNI = 9 | t_(12)_ = -3.3 | p = 0.006 | d = -1.95 |
| 2E - L.gamma | Unpaired t-test | Sham = 5  SNI = 9 | t_(12)_ = -3.27 | p = 0.006 | d = -1.89 |
| 2E - H.gamma | Unpaired t-test | Sham = 5  SNI = 9 | t_(12)_ = -2.07 | p = 0.06 | d = -1.04 |
| 2F - Delta | rank sum test | Sham = 6  SNI = 8 | W = 46 | p = 0.02 | d = 1.38 |
| 2F - Theta | rank sum test | Sham = 6  SNI = 8 | W = 3 | p = 0.002 | d = -1.66 |
| 2F - Sigma | rank sum test | Sham = 6  SNI = 8 | W = 13 | p = 0.11 | d = -0.96 |
| 2F - Beta | rank sum test | Sham = 6  SNI = 8 | W = 13 | p = 0.11 | d = -1 |
| 2F - L.gamma | rank sum test | Sham = 6  SNI = 8 | W = 12 | p = 0.08 | d = -0.99 |
| 2F - H.gamma | rank sum test | Sham = 6  SNI = 8 | W = 11 | p = 0.06 | d = -0.9 |
| 2G | Mixed-model ANOVA | Sham = 6  SNI = 8 | Treatment:  F_(1,13)_ = 3.59  Frequency bands:  F_(5,65)_ = 1145.13  Interaction:  F_(5,65)_ = 5.76 | Treatment:  p = 0.08  Frequency bands:  p = 2x10^-16^  Interaction:  p = 0.008 | Treatment:  f = 0.53  Frequency bands:  f = 9.39  Interaction:  f = 0.51 |
| 2G - Delta | Unpaired t-test | Sham = 5  SNI = 9 | t_(13)_ = 3.13 | p = 0.007 | d = 1.6 |
| 2G - Theta | Unpaired t-test | Sham = 5  SNI = 9 | t_(13)_ = -3.17 | p = 0.007 | d = -1.61 |
| 2G - Sigma | Unpaired t-test | Sham = 5  SNI = 9 | t_(13)_ = -1.59 | p = 0.13 | d = -0.84 |
| 2G - Beta | Unpaired t-test | Sham = 5  SNI = 9 | t_(13)_ = -1.98 | p = 0.06 | d = -1.02 |
| 2G - L.gamma | Unpaired t-test | Sham = 5  SNI = 9 | t_(13)_ = -1.6 | p = 0.13 | d = -0.78 |
| 2G - H.gamma | Unpaired t-test | Sham = 5  SNI = 9 | t_(13)_ = -1.4 | p = 0.17 | d = -0.75 |
|  |  |  |  |  |  |
| 3A - Lightphase  Wake | Mixed-model ANOVA | Sham = 18  SNI = 18 | Treatment:  F_(1,34)_ = 0.004  Day:  F_(1,34)_ = 7.22  Interaction:  F_(1,34)_ = 0.94 | Treatment:  p = 0.9  Day:  p = 0.011  Interaction:  p = 0.33 | Treatment:  f = 0.01  Day:  f = 0.46  Interaction:  f = 0.17 |
| 3A - lightphase  NREMS | Mixed-model ANOVA | Sham = 18  SNI = 18 | Treatment:  F_(1,34)_ = 0.048  Day:  F_(1,34)_ = 12.2  Interaction:  F_(1,34)_ = 2.5 | Treatment:  p = 0.8  Day:  p = 0.0013  Interaction:  p = 0.12 | Treatment:  f = 0.04  Day:  f = 0.6  Interaction:  f = 0.27 |
| 3A - lightphase  REMS | Mixed-model ANOVA | Sham = 18  SNI = 18 | Treatment:  F_(1,34)_ = 0.6  Day:  F_(1,34)_ = 1.77  Interaction:  F_(1,34)_ = 1.46 | Treatment:  p = 0.44  Day:  p = 0.19  Interaction:  p = 0.23 | Treatment:  f = 0.13  Day:  f = 0.23  Interaction:  f = 0.21 |
| 3A - Darkphase  Wake | Mixed-model ANOVA | Sham = 18  SNI = 18 | Treatment:  F_(1,34)_ = 0.79  Day:  F_(1,34)_ = 21.4  Interaction:  F_(1,34)_ = 0.72 | Treatment:  p = 0.79  Day:  p = 5.2x10^-5^  Interaction:  p = 0.72 | Treatment:  f = 0.04  Day:  f = 0.79  Interaction:  f = 0.06 |
| 3A - Darkphase  NREMS | Mixed-model ANOVA | Sham = 18  SNI = 18 | Treatment:  F_(1,34)_ = 0.009  Day:  F_(1,34)_ = 27.4  Interaction:  F_(1,34)_ = 0.37 | Treatment:  p = 0.92  Day:  p = 8.5x10^-6^  Interaction:  p = 0.54 | Treatment:  f = 0.02  Day:  f = 0.9  Interaction:  f = 0.11 |
| 3A - Darkphase REMS | Mixed-model ANOVA | Sham = 18  SNI = 18 | Treatment:  F_(1,34)_ = 1.56  Day:  F_(1,34)_ = 0.11  Interaction:  F_(1,34)_ = 0.62 | Treatment:  p = 0.22  Day:  p = 0.73  Interaction:  p = 0.43 | Treatment:  f = 0.21  Day:  f = 0.06  Interaction:  f = 0.14 |
| 3B - NREMS | Mixed-model ANOVA | Sham = 18  SNI = 18 | Treatment:  F_(1,34)_ = 1.3  Bout Length:  F_(2,68)_ = 206.49  Interaction:  F_(2,68)_ = 0.23 | Treatment:  p = 0.26  Bout Length:  p = 2x10^-16^  Interaction:  p = 0.79 | Treatment:  f = 0.2  Bout Length:  f = 2.46  Interaction:  f = 0.08 |
| 3B - REMS | Mixed-model ANOVA | Sham = 18  SNI = 18 | Treatment:  F_(1,34)_ = 0.09  Bout Length:  F_(2,68)_ = 208.83  Interaction:  F_(2,68)_ = 0.48 | Treatment:  p = 0.76  Bout Length:  p = 2x10^-16^  Interaction:  p = 0.23 | Treatment:  f = 0.05  Bout Length:  f = 2.48  Interaction:  f = 0.21 |
| 3C | Mixed-model ANOVA | Sham = 18  SNI = 18 | Treatment:  F_(1,34)_ = 0.66  Day:  F_(1,34)_ = 0.006  Interaction:  F_(1,34)_ = 0.021 | Treatment:  p = 0.41  Day:  p = 0.94  Interaction:  p = 0.88 | Treatment:  f = 0.14  Day:  f = 0.01  Interaction:  f = 0.02 |
| 3D | Mixed-model ANOVA | Sham = 18  SNI = 18 | Treatment:  F_(1,34)_ = 0.67  Day:  F_(1,34)_ = 0.63  Interaction:  F_(1,34)_ = 0.61 | Treatment:  p = 0.79  Day:  p = 0.43  Interaction:  p = 0.44 | Treatment:  f = 0.04  Day:  f = 0.14  Interaction:  f = 0.13 |
| 3E | Mixed-model ANOVA | Sham = 17  SNI = 14 | Treatment:  F_(1,29)_ = 0.5  Day:  F_(1,29)_ = 9.8  State:  F_(1,29)_ = 70.42  Interaction:  F_(1,29)_ = 5.3 | Treatment:  p = 0.47  Day:  p = 0.004  State:  p = 3x10^-9^  Interaction:  p = 0.027 | Treatment:  f = 0.13  Day:  f = 0.58  State:  f = 1.56  Interaction:  f = 0.43 |
| 3E | Paired t-test  NREMS | Sham = 17 | t_(16)_ = -0.4 | p = 0.69 | d = -0.08 |
| 3E | Paired t-test  NREMS | SNI = 14 | t_(13)_ = -3.75 | p = 0.002 | d = -0.95 |
| 3E | Paired t-test  REMS | Sham = 17 | t_(16)_ = -1.9 | p = 0.07 | d = -0.36 |
| 3E | Paired t-test  NREMS | SNI = 14 | t(13) = -2.47 | p = 0.02 | d = -0.7 |
| 3F | One sample t-test | Sham = 17 | t_(16)_ = 0.97 | p = 0.34 | mean = 0.02 |
| 3F | One sample t-test | SNI = 17 | t_(16)_ = 2.96 | p =0.009 | mean = 0.06 |
| 3G | One sample t-test | Sham = 17 | t_(16)_ = 0.46 | p = 0.64 | mean = 0.009 |
| 3G | One sample t-test | SNI = 17 | t_(16)_ = 2.62 | p = 0.01 | mean = 0.05 |
|  |  |  |  |  |  |
| 4D | Mixed-model ANOVA | Sham = 18  SNI = 18 | Treatment:  F_(1,34)_ = 0.012  Day:  F_(1,34)_ = 0.003  Interaction:  F_(1,34)_ = 2.17 | Treatment:  p = 0.91  Day:  p = 0.95  Interaction:  p = 0.14 | Treatment:  f = 0.02  Day:  f = 0.008  Interaction:  f = 0.25 |
| 4E | 95 CI | Sham = 18  SNI = 18 | Sham: 152.2 ±1.3  SNI: 150.4 ±1.3 | / | / |
| 4F | Mixed-model ANOVA | Sham = 18  SNI = 18 | Treatment:  F_(1,34)_ = 0.046  State:  F_(2,68)_ = 636.9  Interaction:  F_(2,68)_ = 0.6 | Treatment:  p = 0.83  State:  p = 2x10^-16^  Interaction:  p = 0.54 | Treatment:  f = 0.04  State:  f = 4.33  Interaction:  f = 0.13 |
| 4H | One sample t-test | Sham = 12 | t_(16)_ = 17.2 | p = 2.6x10^-9^ | mean = 166.7 |
| 4H | One sample t-test | SNI = 12 | t_(16)_ = 16.5 | p = 4.2x10^-9^ | mean = 170.5 |
| 4H | Unpaired t-test | Sham = 12  SNI = 12 | t_(22)_ = -0.64 | p = 0.52 | d = -0.26 |
| 4J | Mixed-model ANOVA | Sham = 12  SNI = 12 | Treatment:  F_(1,22)_ = 0.9  SD:  F_(1,22)_ = 58.52  Interaction:  F_(1,22)_ = 0.13 | Treatment:  p = 0.35  SD:  p = 1.3x10^-7^  Interaction:  p = 0.72 | Treatment:  f = 0.2  SD:  f = 1.63  Interaction:  f = 0.08 |
| 4L | Mixed-model ANOVA | Sham = 12  SNI = 12 | Treatment:  F_(1,22)_ = 3.08  SD:  F_(1,22)_ = 1.45  Interaction:  F_(1,22)_ = 5.7 | Treatment:  p = 0.09  SD:  p = 0.24  Interaction:  p = 0.025 | Treatment:  f = 0.37  SD:  f = 0.26  Interaction:  f = 0.51 |
| 4L | Paired t-test | Sham = 12 | t_(11)_ = 2.22 | p = 0.048 | d = 0.92 |
| 4L | Paired t-test | SNI = 12 | t_(11)_ = -1.01 | p = 0.33 | d = -0.29 |
| 4L - Control | Unpaired t-test | Sham = 12  SNI = 12 | t_(22)_ = 0.15 | p = 0.88 | d = 0.06 |
| 4L - SD | Unpaired t-test | Sham = 12  SNI = 12 | t_(22)_ = -2.3 | p = 0.03 | d = -0.95 |
|  |  |  |  |  |  |
| 5C - Delta | Mixed-model ANOVA | Sham = 5  SNI = 9 | Treatment:  F_(1,12)_ = 7.16  Period:  F_(1,12)_ = 18.51  Interaction:  F_(1,12)_ = 18.31 | Treatment:  p = 0.02  Period:  p = 0.001  Interaction:  p = 0.001 | Treatment:  f = 0.77  Period:  f = 1.24  Interaction:  f = 1.24 |
| 5C - Delta | Paired t-test | Sham = 5 | t_(4)_ = -7.9 | p = 0.001 | d = -1.02 |
| 5C - Delta | Paired t-test | SNI = 9 | t_(8)_ = -0.8 | p = 0.44 | d = -0.08 |
| 5C - Delta  Continuity | Unpaired t-test | Sham = 5  SNI = 9 | t_(12)_ = 2.11 | p = 0.05 | d = 1.24 |
| 5C - Delta Fragility | Unpaired t-test | Sham = 5  SNI = 9 | t_(12)_ = 3.13 | p = 0.008 | d = 1.84 |
| 5D - Beta | Mixed-model ANOVA | Sham = 5  SNI = 9 | Treatment:  F_(1,12)_ = 9.32  Period:  F_(1,12)_ = 270  Interaction:  F_(1,12)_ = 1 | Treatment:  p = 0.01  Period:  p = 1.36 x10^-9^  Interaction:  p = 0.33 | Treatment:  f = 0.88  Period:  f = 4.74  Interaction:  f = 0.29 |
| 5E - Low gamma | Mixed-model ANOVA | Sham = 5  SNI = 9 | Treatment:  F_(1,12)_ = 11.49  Period:  F_(1,12)_ = 398.3  Interaction:  F_(1,12)_ = 0.75 | Treatment:  p = 0.005  Period:  p = 1.43 x10^-10^  Interaction:  p = 0.4 | Treatment:  f = 0.98  Period:  f = 5.76  Interaction:  f = 0.25 |
| 5F - High gamma | Mixed-model ANOVA | Sham = 5  SNI = 9 | Treatment:  F_(1,12)_ = 3.31  Period:  F_(1,12)_ = 94.3  Interaction:  F_(1,12)_ = 5.83 | Treatment:  p = 0.09  Period:  p = 4.89 x10^-7^  Interaction:  p = 0.03 | Treatment:  f = 0.53  Period:  f = 2.8  Interaction:  f = 0.7 |
| 5F - High gamma | Paired t-test | Sham = 5 | t_(4)_ = 14.17 | p = 0.0001 | d = 0.75 |
| 5F - High gamma | Paired t-test | SNI = 9 | t_(8)_ = 5.45 | p = 0.0006 | d = 0.35 |
| 5F - High gamma  Continuity | Unpaired t-test | Sham = 5  SNI = 9 | t_(12)_ = -1.55 | p = 0.14 | d = -0.91 |
| 5F - High gamma Fragility | Unpaired t-test | Sham = 5  SNI = 9 | t_(12)_ = -2.08 | p = 0.05 | d = -1.2 |
| 5G - Delta | Mixed-model ANOVA | Sham = 6  SNI = 8 | Treatment:  F_(1,12)_ = 0.42  Period:  F_(1,12)_ = 11.9  Interaction:  F_(1,12)_ = 0.009 | Treatment:  p = 0.52  Period:  p = 0.004  Interaction:  p = 0.92 | Treatment:  f = 0.19  Period:  f = 1  Interaction:  f = 0.03 |
| 5H - Beta | Mixed-model ANOVA | Sham = 6  SNI = 8 | Treatment:  F_(1,12)_ = 0.56  Period:  F_(1,12)_ = 127.49  Interaction:  F_(1,12)_ = 0.65 | Treatment:  p = 0.46  Period:  p = 9.5x10^-8^  Interaction:  p = 0.43 | Treatment:  f = 0.22  Period:  f = 3.26  Interaction:  f = 0.23 |
| 5I - Low gamma | Mixed-model ANOVA | Sham = 6  SNI = 8 | Treatment:  F_(1,12)_ = 0.63  Period:  F_(1,12)_ = 75.84  Interaction:  F_(1,12)_ = 0.93 | Treatment:  p = 0.44  Period:  p = 1.56x10^-6^  Interaction:  p = 0.35 | Treatment:  f = 0.23  Period:  f = 2.51  Interaction:  f = 0.28 |
| 5J - High gamma | Mixed-model ANOVA | Sham = 6  SNI = 8 | Treatment:  F_(1,12)_ = 0.004  Period:  F_(1,12)_ = 9.4  Interaction:  F_(1,12)_ = 2.66 | Treatment:  p = 0.94  Period:  p = 0.009  Interaction:  p = 0.12 | Treatment:  f = 0.02  Period:  f = 0.89  Interaction:  f = 0.47 |
|  |  |  |  |  |  |
| 6B | Mixed-model ANOVA | Sham = 5  SNI = 9 | Treatment:  F_(1,12)_ = 8.9  Period:  F_(1,12)_ = 470.6  Interaction:  F_(1,12)_ = 5.33 | Treatment:  p = 0.01  Period:  p = 5.39x10^-11^  Interaction:  p = 0.03 | Treatment:  f = 0.86  Period:  f = 6.26  Interaction:  f = 0.67 |
| 6B | Paired t-test | Sham = 5 | t_(4)_ = 17.98 | p = 5.6x10^-5^ | d = 2.28 |
| 6B | Paired t-test | SNI = 9 | t_(8)_ = 14.8 | p = 4.13x10^-7^ | d = 1.49 |
| 6B - Continuity | Unpaired t-test | Sham = 5  SNI = 9 | t_(12)_ = -2.51 | p = 0.02 | d = -1.4 |
| 6B - Fragility | Unpaired t-test | Sham = 5  SNI = 9 | t_(12)_ = -3.44 | p = 0.004 | d = -1.97 |
| 6C | Mixed-model ANOVA | Sham = 5  SNI = 9 | Treatment:  F_(1,12)_ = 0.2  Period:  F_(1,12)_ = 3.83  Interaction:  F_(1,12)_ = 1.31 | Treatment:  p = 0.65  Period:  p = 0.07  Interaction:  p = 0.27 | Treatment:  f = 0.13  Period:  f = 0.57  Interaction:  f = 0.33 |
| 6E | Mixed-model ANOVA | Sham = 5  SNI = 9 | Treatment:  F_(1,12)_ = 11.26  Period:  F_(1,12)_ = 25.3  Interaction:  F_(1,12)_ = 2.6 | Treatment:  p = 0.005  Period:  p = 0.0003  Interaction:  p = 0.13 | Treatment:  f = 0.97  Period:  f = 1.45  Interaction:  f = 0.47 |
| 6F | Mixed-model ANOVA | Sham = 5  SNI = 9 | Treatment:  F_(1,12)_ = 0.045  Period:  F_(1,12)_ = 44.5  Interaction:  F_(1,12)_ = 0.9 | Treatment:  p = 0.83  Period:  p = 2.3x10^-5^  Interaction:  p = 0.35 | Treatment:  f = 0.06  Period:  f = 1.93  Interaction:  f = 0.28 |
| 6H | Mixed-model ANOVA | Sham = 5  SNI = 9 | Treatment:  F_(1,12)_ = 1.75  Period:  F_(1,12)_ = 11.19  Peak or no peak:  F_(1,12)_ = 26.7  peak x period:  F_(1,12)_ = 10.67 | Treatment:  p = 0.21  Period:  p = 0.005  Peak or no peak:  p = 0.0002  peak x period:  p = 0.006 | Treatment:  f = 0.38  Period:  f = 0.97  Peak or no peak:  f = 1.49  peak x period:  f = 0.94 |
| 6H - Continuity | Paired t-test peak vs no-peak | Sham = 5 | t_(4)_ = -4.01 | p = 0.015 | d = -1.6 |
| 6H - Fragility | Paired t-test peak vs no-peak | Sham = 5 | t_(4)_ = -4.61 | p = 0.009 | d = -1.8 |
| 6H - Continuity | Paired t-test peak vs no-peak | SNI = 9 | t_(8)_ = -2.8 | p = 0.02 | d = -1.31 |
| 6H - Fragility | Paired t-test peak vs no-peak | SNI = 9 | t_(8)_ = -3.5 | p = 0.007 | d = -1.91 |
| 6I | Unpaired t-test | Sham = 5  SNI = 9 | t_(12)_ = -2.6 | p = 0.02 | d = -1.62 |
|  |  |  |  |  |  |
| 8D | Mixed-model ANOVA | Sham = 5  SNI = 9 | Treatment:  F_(1,12)_ = 11.07  Period:  F_(1,12)_ = 136.6  Interaction:  F_(1,12)_ = 0.3 | Treatment:  p = 0.005  Period:  p = 1.31x10^-8^  Interaction:  p = 0.58 | Treatment:  f = 0.89  Period:  f = 3.12  Interaction:  f = 0.15 |
|  |  |  |  |  |  |
| SUp 2B | Mixed-model ANOVA | Sham = 5  SNI = 9 | Treatment:  F_(1,12)_ = 14.9  MAs:  F_(1,12)_ = 110.2  Interaction:  F_(1,12)_ = 0.76 | Treatment:  p = 0.002  MAs:  p = 2.1x10^-7^  Interaction:  p = 0.39 | Treatment:  f = 1.12  MAs:  f = 3.03  Interaction:  f = 0.25 |
| SUp 2C | Mixed-model ANOVA | Sham = 5  SNI = 9 | Treatment:  F_(1,12)_ = 0.24  MAs:  F_(1,12)_ = 101.6  Interaction:  F_(1,12)_ = 1.39 | Treatment:  p = 0.62  Period:  p = 3.28x10^-7^  Interaction:  p = 0.26 | Treatment:  f = 1.14  MAs:  f = 2.91  Interaction:  f = 0.34 |
| SUp 2D - Left | Wilcoxon rank-sum | Sham = 4  SNI = 6 | W = 10 | p = 0.76 | d = -0.006 |
| SUp 2D - Middle | Wilcoxon signed-rank  MAs | Sham = 4  SNI = 6 | V = 10 | p = 0.12 | d = 1.55 |
| SUp 2D - Middle | Wilcoxon signed-rank  NREMS | Sham = 4  SNI = 6 | V = 21 | p = 0.03 | d = 1.86 |
| SUp 2D - Right | Mixed-model ANOVA | Sham = 5  SNI = 9 | Treatment:  F_(1,8)_ = 2.06  MAs:  F_(1,8)_ = 39.12  Interaction:  F_(1,8)_ = 1.39 | Treatment:  p = 0.18  MAs:  p = 0.0002  Interaction:  p = 0.36 | Treatment:  f = 0.51  MAs:  f = 2.21  Interaction:  f = 0.34 |
| SUp 2E - Left | Unpaired t-test | Sham = 10  SNI = 12 | t_(21)_ = -0.5 | p = 0.62 | d = 0.004 |
| SUp 2E - Middle | Mixed-model ANOVA | Sham = 10  SNI = 12 | Treatment:  F_(1,8)_ = 0.8  MAs:  F_(1,8)_ = 446.8  Interaction:  F_(1,8)_ = 1.6 | Treatment:  p = 0.37  MAs:  p = 1.23x10^-15^  Interaction:  p = 0.2 | Treatment:  f = 0.2  MAs:  f = 4.61  Interaction:  f = 0.28 |
| SUp 2E - Right | Mixed-model ANOVA | Sham = 10  SNI = 12 | Treatment:  F_(1,8)_ = 0.29  MAs:  F_(1,8)_ = 174.9  Interaction:  F_(1,8)_ = 0.03 | Treatment:  p = 0.59  MAs:  p = 1.18x10^-11^  Interaction:  p = 0.85 | Treatment:  f = 0.12  MAs:  f = 2.89  Interaction:  f = 0.04 |
| SUp 2F - Left | Unpaired t-test | Sham = 6  SNI = 8 | t_(12)_ = -0.23 | p = 0.82 | d = 0.003 |
| SUp 2F - Middle | Mixed-model ANOVA | Sham = 6  SNI = 8 | Treatment:  F_(1,12)_ = 0.89  MAs:  F_(1,12)_ = 63.9  Interaction:  F_(1,12)_ = 0.54 | Treatment:  p = 0.36  MAs:  p = 3.77x10^-6^  Interaction:  p = 0.47 | Treatment:  f = 0.27  MAs:  f = 2.31  Interaction:  f = 0.21 |
| SUp 2F - Right | Mixed-model ANOVA | Sham = 6  SNI = 8 | Treatment:  F_(1,12)_ = 1.8  MAs:  F_(1,12)_ = 50.26  Interaction:  F_(1,12)_ = 1.7 | Treatment:  p = 0.19  Period:  p = 1.27x10^-5^  Interaction:  p = 0.21 | Treatment:  f = 0.39  MAs:  f = 2.05  Interaction:  f = 0.38 |
