# Supplementary material for: Cortico-autonomic local arousals and heightened somatosensory arousability during NREMS of mice in neuropathic pain
Source: eLife. 2021 Jul 6;10:e65835. doi: 10.7554/eLife.65835 (PMC8291975; doi:10.7554/eLife.65835)
Supplement: Transparent reporting form [file elife-65835-transrepform1.docx]

***eLife’s* transparent reporting form**

We encourage authors to provide detailed information *within their submission* to facilitate the interpretation and replication of experiments. Authors can upload supporting documentation to indicate the use of appropriate reporting guidelines for health-related research (see [EQUATOR Network](http://www.equator-network.org/%20)), life science research (see the [BioSharing Information Resource](https://biosharing.org/)), or the [ARRIVE guidelines](http://www.plosbiology.org/article/info:doi/10.1371/journal.pbio.1000412) for reporting work involving animal research. Where applicable, authors should refer to any relevant reporting standards documents in this form.

If you have any questions, please consult our Journal Policies and/or contact us: [editorial@elifesciences.org](mailto:editorial@elifesciences.org).

**Sample-size estimation**

- You should state whether an appropriate sample size was computed when the study was being designed
- You should state the statistical method of sample size computation and any required assumptions
- If no explicit power analysis was used, you should describe how you decided what sample (replicate) size (number) to use

Please outline where this information can be found within the submission (e.g., sections or figure legends), or explain why this information doesn’t apply to your submission:

The number of animals/recordings per group was determined with the resource equation method from (Charan and Kantharia, 2013). No otherwise explicit power analysis was performed. For all experiments involving Sham and SNI animals, we planned for an E value between 10 to 20 and the lowest we reached was 10, after animal exclusion as a result of post-mortem histological analysis. Previous baseline recordings from the lab were included in Figure 1 for optimal representation of microarousal distributions in the mouse line used and in Figure 7 for independence in the training of the network.

**Replicates**

- You should report how often each experiment was performed
- You should include a definition of biological versus technical replication
- The data obtained should be provided and sufficient information should be provided to indicate the number of independent biological and/or technical replicates
- If you encountered any outliers, you should describe how these were handled
- Criteria for exclusion/inclusion of data should be clearly stated
- High-throughput sequence data should be uploaded before submission, with a private link for reviewers provided (these are available from both GEO and ArrayExpress)

Please outline where this information can be found within the submission (e.g., sections or figure legends), or explain why this information doesn’t apply to your submission:

1. We report all animal numbers used for the different experiments in the figures and in the main text. We also list the total numbers of animals used in the first paragraph of the methods. Additionally, we indicate how often each experiment was performed in there.
2. The biological replicate are the animals, and in the case of figure 1D, the microarousals (MAs) from 30 animals. This latter measure reached significance in any individual animals with very low inter-individual variability, therefore we used the MAs themselves as biological replicates. For EEG/EMG/LFP recordings, the technical replicates consisted of two 12 to 24 h-long recordings directly following each other, from which the mean of any specific measure was used per animal. Only the sleep deprivation did not have technical replicates as this treatment was applied only once per animal. For the arousability experiment shown in figure 8, we used 2-3 12 h-long experiments per animal per condition.
3. As mentioned, all biological replicate numbers are specified in the figures. Technical replicate numbers are specified in the Methods.
4. We did not treat data as outliers. Datapoints that qualify as outliers in the classical boxplot visualization are shown as grey crosses located beyond the whiskers (as they are 1.5 x larger than the interquartile range) in several figure panels. For all these cases, outliers were always included in all statistical analyses.
5. No data points were excluded. Criteria for exclusion of animals were based on anatomical assessment of the LFP electrode placement, and in the case of heart rate measures, by the quality of the EMG electrode in showing R-R intervals (stated in the legends and methods).
6. N/A

**Statistical reporting**

- Statistical analysis methods should be described and justified
- Raw data should be presented in figures whenever informative to do so (typically when N per group is less than 10)
- For each experiment, you should identify the statistical tests used, exact values of N, definitions of center, methods of multiple test correction, and dispersion and precision measures (e.g., mean, median, SD, SEM, confidence intervals; and, for the major substantive results, a measure of effect size (e.g., Pearson's r, Cohen's d)
- Report exact p-values wherever possible alongside the summary statistics and 95% confidence intervals. These should be reported for all key questions and not only when the p-value is less than 0.05.

Please outline where this information can be found within the submission (e.g., sections or figure legends), or explain why this information doesn’t apply to your submission:

1. The statistics section of the methods describes in detail which tests were used and why. The ANOVA factors and levels are given. Additionally, in each figure legend, it is indicated which statistical test was used.

2. All data points are shown as points overlapping the boxplots, or as black lines between boxplots in case of repeated measures.

3. We add a Supplementary Statistics Table in which all this information is specified for every figure panel. Basic information is additionally stated in the figure legends. In the figures, significant p values for ANOVAs and post-hoc tests are written directly next to the axes and next to the datasets, respectively.

4. The complete set of these values is found in the Supplementary Statistics Table. Additionally, the main text, figures and figure legends contain these values as follows: Main text: Values for n, significant ANOVAs and post-hoc tests; Figures: Values for significant ANOVAs next to the figure axes, values for post hoc tests next to the datasets; Figure legends: types of statistical tests used; Statistical Methods: Choice of statistical tests, factors used in ANOVAs.

(For large datasets, or papers with a very large number of statistical tests, you may upload a single table file with tests, Ns, etc., with reference to sections in the manuscript.)

**Group allocation**

- Indicate how samples were allocated into experimental groups (in the case of clinical studies, please specify allocation to treatment method); if randomization was used, please also state if restricted randomization was applied
- Indicate if masking was used during group allocation, data collection and/or data analysis

Please outline where this information can be found within the submission (e.g., sections or figure legends), or explain why this information doesn’t apply to your submission:

Our animals were randomly assigned to the Sham or SNI groups by the technician performing the surgeries. The experimenter was blind to the treatment during recordings and analysis until figures had to be prepared. For the case of LFP recordings, analysis was blind until the decision for inclusion or exclusion was made based on histological verification.

**Additional data files (“source data”)**

- We encourage you to upload relevant additional data files, such as numerical data that are represented as a graph in a figure, or as a summary table
- Where provided, these should be in the most useful format, and they can be uploaded as “Source data” files linked to a main figure or table
- Include model definition files including the full list of parameters used
- Include code used for data analysis (e.g., R, MatLab)
- Avoid stating that data files are “available upon request”

Please indicate the figures or tables for which source data files have been provided:

Every data point is represented in the figures. We provide csv files named after each figure panel containing the numerical data. We also provide all codes used for analysis.
